# Nanoceria: A Rare-Earth Nanoparticle as a Novel Anti-Angiogenic Therapeutic Agent in Ovarian Cancer

**DOI:** 10.1371/journal.pone.0054578

**Published:** 2013-01-31

**Authors:** Shailendra Giri, Ajay Karakoti, Rondell P. Graham, Jacie L. Maguire, Christopher M. Reilly, Sudipta Seal, Ramandeep Rattan, Viji Shridhar

**Affiliations:** 1 Department of Experimental Pathology, Mayo Clinic College of Medicine, Mayo Clinic, Rochester, Minnesota, United States of America; 2 Mechanical Materials Aerospace Eng, Advanced Materials Processing Analysis Center, Nanoscience Technology Center, University of Central Florida, Orlando, Florida, United States of America; 3 Anatomic/Clinical Pathology, Mayo Clinic College of Medicine, Mayo Clinic, Rochester, Minnesota, United States of America; 4 Via Virginia College of Osteopathic Medicine and Virginia-Maryland Regional College of Veterinary Medicine Virginia Tech, Blacksburg Virginia, United States of America; 5 Women’s Health Services, Henry Ford Health System, Detroit, Michigan, United States of America; University of Nebraska Medical Center, United States of America

## Abstract

Ovarian cancer (OvCa) is the fifth most common cause of death from all cancers among women in United Sates and the leading cause of death from gynecological malignancies. While most OvCa patients initially respond to surgical debulking and chemotherapy, 75% of patients later succumb to the disease. Thus, there is an urgent need to test novel therapeutic agents to counteract the high mortality rate associated with OvCa. In this context, we have developed and engineered Nanoceria (NCe), nanoparticles of cerium oxide, possessing anti-oxidant properties, to be used as a therapeutic agent in OvCa. We show for the first time that NCe significantly inhibited production of reactive oxygen species (ROS) in A2780 cells, attenuated growth factor (SDF1, HB-EGF, VEGF_165_ and HGF) mediated cell migration and invasion of SKOV3 cells, without affecting the cell proliferation. NCe treatment also inhibited VEGF_165_ induced proliferation, capillary tube formation, activation of VEGFR2 and MMP2 in human umbilical vascular endothelial cells (HUVEC). NCe (0.1 mg/kg body weigh) treatment of A2780 ovarian cancer cells injected intra-peritoneally in nude mice showed significant reduction (p<0.002) in tumor growth accompanied by decreased tumor cell proliferation as evident from reduced tumor size and Ki67 staining. Accumulation of NCe was found in tumors isolated from treated group using transmission electron microscopy (TEM) and inductively coupled plasma mass spectroscopy (ICP-MS). Reduction of the tumor mass was accompanied by attenuation of angiogenesis, as observed by reduced CD31 staining and specific apoptosis of vascular endothelial cells. Collectively, these results indicate that cerium oxide based NCe is a novel nanoparticle that can potentially be used as an anti-angiogenic therapeutic agent in ovarian cancer.

## Introduction

In the United States, 27,000 women are newly diagnosed and approximately 14, 000 women die from OvCa annually [1]. Such high mortality rates are due to majority of patients (75%) presenting with advanced (stage III or greater) disease at the time of diagnosis [2]. More than 90% of the patients have better prognosis if the cancer is detected in its earliest stages. Treatment of epithelial ovarian cancer generally involves surgical debulking followed by chemotherapy with a combination of platinum and a taxane-containing agent. However, majority of patients recur and ultimately succumb to their cancer. Consequently, there is an urgent need to develop new therapeutics that can be more effective in treating ovarian cancer and delaying or preventing recurrences. Novel therapies that target ovarian tumorigenesis are extensively been researched, but we have yet to come up with a promising drug.

Nanotechnology based tools and techniques are rapidly emerging in the fields of medical imaging and targeted drug delivery. Cerium oxide is a rare-earth oxide that is found in the lanthanide series of the periodic table. Nanocrystalline cerium oxide (nanoceria) exhibits a blue shift in the ultraviolet absorption spectrum, the shifting and broadening of Raman allowed modes and lattice expansion as compared to bulk cerium oxide indicating its unique properties. NCe has emerged as a lucrative material in biomedical science due to its unique ability to switch oxidation states between (III) and (IV) depending upon the environment. The ability to switch between mixed oxidation states of nanoceria is comparable to biological antioxidants. This imparts nanoceria with a very important biological property of radical scavenging which can be tuned based upon the retention of oxygen vacancies (defects) and concentration of Ce3+ species in nanoceria. The reversibility of oxidation state is the key property in making nanoceria a potent antioxidant, thereby reducing the need for frequent repeated dosage. Previous studies have demonstrated that cerium oxide nanoparticles possess excellent antioxidant properties and act as potent, regenerative free radical scavengers in biological systems [3], [4], [5]. These regenerative antioxidant properties are due, in part, to the valence structure of the cerium atom combined with inherent defects in the crystal lattice structure, which are magnified at the nano-scale. It has been suggested that the unique structure of engineered cerium oxide nanoparticles, with respect to valence and oxygen defects, promotes cell longevity and decreases toxic insults by virtue of its antioxidant effects that occur when the nanoparticles enter the cells [6], preventing the accumulation of reactive oxygen species (ROS) in the cell [3].

Tumor angiogenesis is characterized by the formation of new irregular blood vessels from a preexisting vascular network. This abnormal angiogenesis is required for the growth, survival, and metastasis of most solid tumors [7], [8]. Vascular endothelial growth factor (VEGF) is one of the most important pro-angiogenic factors, which acts as a mitogen for vascular endothelial cells *in vitro* and as an angiogenic factor *in vivo* [9]. It is over expressed in various human cancers [10], [11], [12], [13] including OvCa. Recently, it has been suggested that ROS plays an important role in regulating tumor induced angiogenesis by controlling VEGF production. Enhanced production of VEGF has been shown to correlate with a poor outcome for patients with both early and advanced OvCa. Various anti-angiogenic agents have been and are undergoing evaluations in ovarian cancer clinical trials. A phase II study of single-agent bevacizumab (a monoclonal antibody directed against VEGF) showed promising results [14]. Therefore, VEGF signaling is becoming the focus of anti-angiogenic-targeted therapy in OvCa.

In the present study, we have tested cerium oxide nanoparticles as a therapeutic agent both *in* *vitro* and *in vivo* in OvCa cells. Our data demonstrates that NCe was able to inhibit growth factor mediated, migration and invasion of SKOV3 cells, VEGF_165_ induced proliferation, capillary tube formation and activation of VEGFR2 and MMP2 in HUVEC cells. More importantly NCe treatment inhibited tumor growth *in vivo* by inhibiting angiogenesis, specifically by targeting vascular endothelial cells.

## Materials and Methods

### Reagents and Antibodies

Trypan Blue, MTT 3-(4,5-dimethylthiazol-2-yl)-2,5-diphenyl tetrazolium bromide) and HB-EGF were from Sigma. SDF1, VEGF_165_ and HGF were purchased from R&D Systems (MN, USA). Ki-67 and VEGF antibodies were from Dako (Glostrup, Denmark) and Abcam (MA, USA) respectively. CD31 (PECAM) was from Santa Cruz Biotechnology (CA, USA).

### Cell Culture

Human ovarian cancer cell line SKOV3 and HUVEC were from American Type Culture Collection. A2780 and C200 cell lines were a kind gift of Dr. Tom Hamilton (Fox Chase Cancer Center). OvCa cell lines were maintained and cultured in complete RPMI media containing 10% FBS, antibiotics. HUVEC cells were maintained in EBM-2 media purchased from Lonza (Denmark).

### Nanoparticle Synthesis

Cerium Oxide nanoparticles were prepared by wet chemical synthesis as previously described [15], [16], [17]. Briefly, cerium nitrate hexahydrate was dissolved in deionized water and then filtered using a 200 nm filter to get rid of any freely suspending particulates. The solution containing cerium ions was then oxidized using hydrogen peroxide and ammonium hydroxide. The pH of the solution was adjusted between 3.5–4.0 by using nitric acid or ammonium hydroxide. All the glassware were autoclaved before being used for synthesis. The pH and the zeta potential of the suspension was closely monitored as the solution was allowed age at room temperature for next several days until the formation of nanoparticles with predominantly Ce^3+^ concentration was observed using UV-Visible spectrophotometry.

### Nanoparticles Characterization

Change in the oxidation states of the as-prepared nanoparticles in solution was monitored using UV-Visible spectrophotometry. Aliquots from the parent sample were taken for absorbance measurements by using Perkin Elmer 750 S spectrophotometer. The particle morphology and size distribution was studied using high-resolution transmission electron microscopy (HRTEM). The size of the nanoparticles in as prepared solution prior to use in *in-vitro* and *in-vivo* studies was also monitored using dynamic light scattering (DLS). The oxidation states of cerium inthe particles were confirmed using X-ray photoelectron spectroscopy (XPS). For High Resolution Transmission Electron Microscopy (HRTEM) a drop of suspension of nanoparticles was casted on the carbon-coated copper grid. The HRTEM images of the as-prepared particles were obtained with a Philips (Tecnai Series) operated at 300 keV. The XPS data were obtained using a 5400 PHI ESCA (XPS) spectrometer. Samples were drop casted on a silicon wafer and dried inside a nitrogen glove box to avoid the oxidation of cerium from atmospheric oxygen and transferred using a sample transfer chamber without exposing the samples to atmosphere. Only limited scans were obtained to avoid x-ray damage of cerium. An initial scan was saved separately to compare with the combined 5 scan results and showed no difference in the Ce^3+^/Ce^4+^ ratio. The base pressure during XPS analysis was 10^−9^ Torr and Mg-K*_α_* X-ray radiation (1253.6 eV) at a power of 200 W was used as x-ray source. The binding energy of Au (4f_7/2_) at 84.0±0.1 eV was used to calibrate the binding energy scale of the spectrometer. Any charging shift produced in the spectrum was corrected by referencing to the C (1 s) position at (284.6 eV) [13]. XPS spectra smoothening and baseline subtraction was carried out using PeakFit (Version 4) software.

### Migration and Invasion Assays

SKOV3 cells were grown in serum-free media overnight. Cell migration and invasion were measured as described [18] with modifications. Briefly, cell suspensions (500 µl, 2.5×10^4^ cells) were seeded on the top of uncoated (migration assay) and Matrigel-coated (invasion assay) transwell plates (8-µm pore diameter; BD Biosciences). Serum-free cell suspensions (500 µl) were added to the top chamber of the transwell. The lower chambers contained serum free media containing various growth factors including SDF1, HB-EGF, VEGF_165_ and HGF at the concentration of 25 ng/ml. Cells invading the lower chamber were stained with 0.5% crystal violet (60% PBS, 40% EtOH) and counted with an inverted microscope. The results from at least two independent experiments in triplicate are presented.

### Proliferation Assays

*(i) MTT assay:* 2.5–5.0×10^4^ cells were plated in 24 well plates in triplicates and treated with indicated concentrations of NCe for 72 h. MTT assay was performed as described before [19], to ascertain the number of live cells. *(ii) Thymidine incorporation:* Proliferation of cells was also determined by [^3^H]-thymidine incorporation into DNA as described before [20]. In brief, 2.5–5.0×10^4^ cells were plated in 24 well plates in triplicates and treated with indicated concentrations of NCe for 72 h. Each group was treated with 1 µCi of [^3^H]thymidine in the same medium for 6 h. The adherent cells were fixed by 5% trichloroacetic acid and lysed in SDS/NaOH lysis buffer. Radioactivity was measured by Beckman LS3801 liquid scintillation counter (Canada).

### Colony Formation Assay

2000 cells were plated in triplicates in 6-well plates and treated with indicated concentrations of NCe. The cells were allowed to form colonies for up to 2–4 weeks (depending on the cell line) and media was replaced every fourth day. Colonies were stained with MTT and counted as described before [19].

### Measurement of ROS

ROS was determined using the membrane-permeable fluorescent dye 6-carboxy 2′, 7′-dichlorodihydrofluorescein diacetate (DCFDA) in serum-free medium as described previously [21], [22]. The cultured cells, with or without treatment with NCe, were treated with 5 µM DCF dye in PBS and change in fluorescence was recorded at excitation 485 nm and emission 530 nm for various time period from 10 to 60 min using a Soft Max Pro spectrofluorometer (Molecular Devices, Sunnyvale, CA).

### Immunoblot Analysis

After stipulated time of incubation in the presence or absence of indicated amounts of NCe, immunoblot analysis with specific antibodies was performed as previously described [19], [20], [21], [22]. In brief, treated and untreated HUVEC cells with VEGF_165_ (25 ng/ml) and/or NCe at various time period (5–30 min) were lysed in lysis buffer (50 mM Tris-HCl (pH 7.5), 250 mM NaCl, 5 mM EDTA, 50 mM NaF, and 0.5% Nonidet P-40] containing a protease inhibitor cocktail (Sigma). 40 µg of proteins were resolved by SDS-PAGE and transferred onto nitrocellulose membrane. The membrane was then blocked for 1 h in 5% nonfat dry milk TTBS (20 mM Tris, 500 mM NaCl, and 0.1% Tween 20, pH 7.5) and incubated overnight in primary antisera (pVEGFR (Y1175), pVEGFR (Y951), VEGFR2 or β actin) containing 5% nonfat dry milk or 5% BSA in case of phospho-antibodies. After incubation with HRP-conjugated secondary Ab, blots were developed with an ECL detection system (GE Healthcare, Piscataway, NJ).

### In Vitro Vascular Tube Formation Assay

*In vitro* tube formation assays were performed as described by Melinda *et. al.* [23]. In brief, matrigel matrix was uniformly plated onto 8-well chamber slides (0.15 ml) and incubated at 37 C for 30 min. 2×10^5^/ml HUVEC cells were treated with NCe (25–50 µM) and mixed with cells in the presence or absence of VEGF_165_ (25 ng/ml) and transferred to each well (200 µl) coated with matrigel. The plates or slides were incubated at 37°C for 16 h and imaged under a phase contrast inverted microscope at 10X objective magnification.

### Zymography Assay

For MMP2 activity, HUVEC cells were treated with VEGF_165_ (25 ng/ml) in the presence or absence of NCe (25–50 µM). Post 18 h, cell supernatant was collected, centrifuged at 12,000 g and 25 µl of volume was mixed with 5× SDS loading buffer without reducing agent and ran on 10% tris-glycine gel containing gelatin. Gel was washed twice for 1 h with renaturating solution (2.5% tritonX100 in 50 mM Tris pH 7.4, 5 mM CaCl, 1 mM ZnCl2) to remove SDS and renature MMPs. After rinsing gel with deionized water, gel was incubated overnight at 37°C with buffer containing 50 mM Tris pH 7.4, 5 mM CaCl, 1 mM ZnCl_2_. Next day, gel was stained with 0.5% Coomassie G250 followed by de-staining to see MMP2 activity in gel.

### Animals

#### Ethics statement

6–8 wk old female nude mice were purchased from the National Cancer Institute-Frederick Cancer Research and Development Center (Frederick, MD). All mice were housed and maintained under specific conditions in facilities at Mayo Clinic Rochester, MN. The facilities are approved and inspected by the American Association for Accreditation of Laboratory Animal Care (AAALAC Accreditation # 000717) and in accordance with current regulations and standards of the U.S. Department of Agriculture, U.S. Department of Health and Human Services, and NIH. All studies were approved and supervised by the Mayo Clinic Institutional Animal Care and Use Committee (IACUC) under the protocol number A11309. After tumor induction, mice were monitored daily for signs of any distress. Regular health checks are also performed by the Mayo veterinarian staff. Mice were humanly sacrificed when the tumor burden reached the mandate weight in untreated mice.

Mice were maintained according to Institutional IACUC-approved protocol. 2×10^6^/200 µl cells suspended in PBS were injected into the intra-peritoneal cavity (day 0) of 6–7 week old nude mice as described before [24]. Mice were randomized into two groups. NCe treatment at the dose of 0.1 mg/kg body weight began 3 days post inoculation of cells and was given intra-peritoneally at every 3^rd^ day till end of the study. The control group received PBS injections. Mice were sacrificed at 4 weeks and tumors were fixed in formalin for sectioning. Blood was collected in heparin coated tubes to obtain plasma. Liver, kidney, heart and spleen from all animals were formalin fixed and processed. One tumor and organ slide from each mouse was stained with H&E.

#### Cytotoxicity assays

Blood was collected in heparin coated tubes just before mice were sacrificed. Plasma isolated from blood of six mice from each group was subjected to analysis of a panel of liver function tests (aspartate aminotransferase; alanine aminotransferase; albumin) and kidney function tests (creatinine; urea; albumin) as described before [24]. All assays were performed using kits from Bioassay Systems following the manufacturer’s instruction (CA, USA).

#### Determination of NCe in tissues

At the end of the study, various tissues including tumor, liver, spleen and kidney from NCe treated group were placed in 70% nitric acid overnight to start the digestion process. Samples were then microwave digested. The temperature was ramped to 200°C over 20 min and held there for another 20 min. Samples were then boiled down to less than 1 ml each and reconstituted in water to an exact volume of 10 ml. Cerium levels were assessed using inductively coupled plasma mass spectroscopy (ICP-MS) as described before [25].

For transmission electron microscopy (TEM), ovarian tumor isolated from NCe treated mice were fixed in Trump’s fixative (1% glutaraldehyde and 4% formaldehyde in 0.1 M phosphate buffer, pH 7.2). Tumor was embedded in Spurr’s resin and thin (90 nm) sections were cut on a Reichert Ultracut E ultramicrotome, placed on 200 mesh copper grids and stained with lead citrate. Micrographs were taken on a TECNAI 12 operating at 120KV.

#### IHC

Fixed tumors excised from mice were processed for immunohistochemistry for CD31, Ki-67 and TUNEL (Millipore) as previously described [24]. H&E staining was performed by Mayo immunohistochemical Core facilities. Ki-67 and H&E sections were examined under light microscope and representative pictograms were taken from 5 different slides of each group. For CD31 staining, TRITC-labeled secondary antibody was used and visualized using fluorescent microscope. For double staining, the slides were first processed for CD31 staining followed by immunofluorescence TUNEL staining, according to manufacturer’s instructions.

#### Live tumor measurements

Maximum diameter of viable tumor was calculated by RPG by summing the largest uni-dimensional diameter of each fragment of tumor using the Olympus BX-41 microscope and a micrometer. Similarly, necrotic areas were measured and the composite live tumor size was calculated from each slide.

#### Endothelial tube formation

To examine the effect of NCe on tube formation in HUVEC cells, matrigel matrix was uniformly plated onto 8-well chamber slides (0.15 ml) and incubated at 37°C for 30 min. HUVEC cells were counted and diluted to 2 × 10^5^/ml in medium. Cells were treated with NCe (25–50 µM) was mixed with cells in the presence or absence of VEGF_165_ (25 ng/ml) and transferred to each well (200 µl) coated with matrigel. The plates or slides were incubated at 37°C for 16 h and imaged under a phase contrast inverted microscope at 10× objective magnification.

#### Statistical analysis

The data were statistically analyzed using two-tailed Student’s t-test (Prism). ***P<0.001; **P<0.01,*P<0.05; NS: not significant compared to untreated.

## Results

### Synthesis and Characterization of Cerium Oxide Nanoparticles

**Figure 1:**
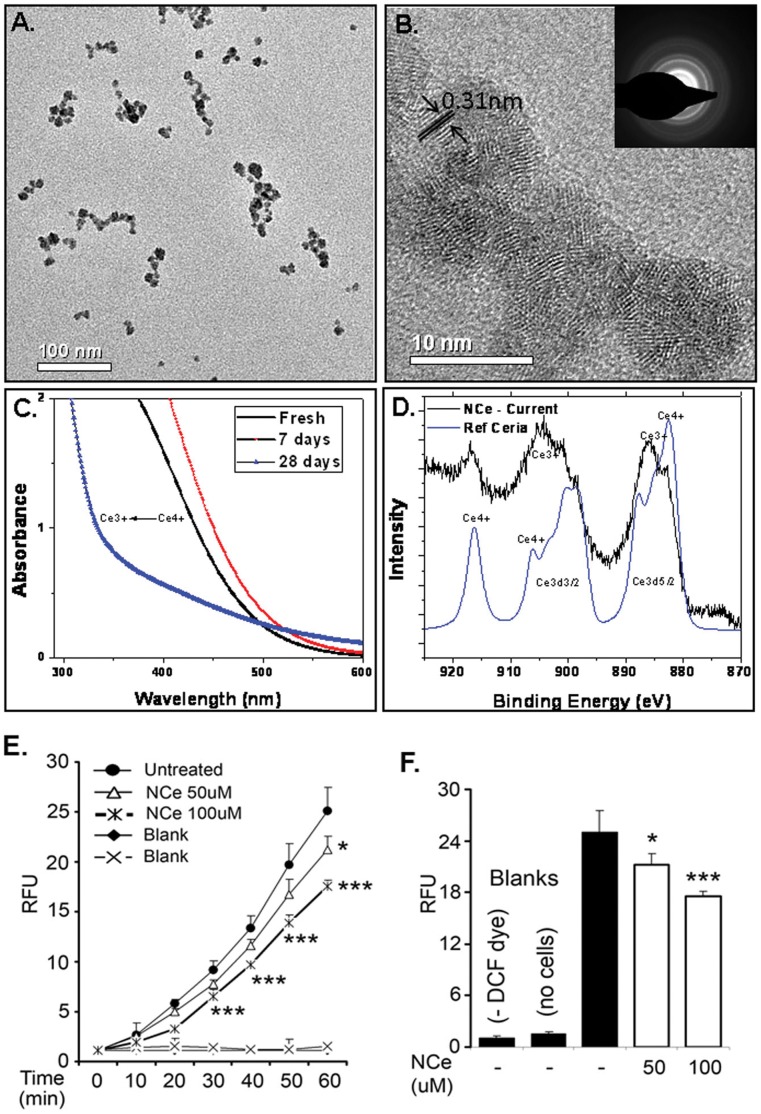
Preparation and characterization of NCe. High resolution transmission electron micrographs show the presence of **A** loose agglomerates of 15–20 nm at low magnification **B** individual 3–5 nm crystallites. The d spacing of 0.31 nm shows the presence of planes of ceria while the selected area electron diffraction (SAED) pattern confirms the presence of fluorite lattice of cerium oxide. The trend in oxidation state of nanoceria in **C** shows that the synthesized nanoparticles have predominance of trivalent oxidation state that undergoes a slow transformation to Ce^+3^ oxidation state over a period of 28 days **D** x-ray photoelectron spectrum from a reference ceria sample in which cerium is predominantly in +4 oxidation state is compared to the spectrum from a 4 weeks aged sample of nanoceria demonstrating the high concentration of Ce in +3 oxidation states. **E.** Basal levels of ROS in ovarian cancer cell line. A2780 cells were treated with NCe (50–100 µM) for 48 h. Cells were washed with PBS and loaded with DCF-DA dye (5 µM) and fluorescence was recorded at excitation 485 nm and emission 530 nm for various time periods (5–60 min). Wells containing only cells without DCFDA dye (cross) or without cells containing DCFDA dye (filled diamond) were used as a blank. **F.** Bar graph represents ROS levels at the 60 min of treatment with DCF-DA dye. Results are shown as mean ± S.D. of 4 samples. ***p<0.001 NCe at 100 uM; *p<0.05 NCe compared to untreated cells using two-tailed Student’s t-test (Prism).

Cerium oxide nanoparticles used in this study contains individual crystallites of 3–5 nm that are loosely agglomerated to 15–25 nm. As the synthesis process is free from any organic surfactant, the hard agglomeration of nanoparticles is controlled by tightly controlling the pH of the nanoparticles below 3.5 during synthesis to keep them in colloidal range. Nanoparticles can be diluted in aqueous or cellular media after the synthesis. Figure 1 A and B shows the high resolution transmission electron micrographs (HRTEM) of NCe nanoparticles. It is evident from the image that nanoparticles are loosely agglomerated to about 15–25 nm aggregates which could also be induced by the drying process. The hydrodynamic radius (37.8 nm ±0.8) from the multimodal size distribution (volume %) analysis of DLS measurements agrees with the loose agglomerate size of the HRTEM analysis. High magnification image confirms the lattice planes of NCe in individual 3–5 nm crystallites. UV-Visible spectroscopy was used to analyze the oxidation states of cerium oxide nanoparticles before and after aging treatment. Figure 1 shows the UV-Visible spectra from fresh and aged cerium oxide nanoparticles clearly indicating the predominance of Ce^+3^ oxidation state from the absorption peak at 252 nm as compared to absorption peak at 298 nm for Ce^4+^. Further confirmation on the oxidation states of nanoparticles was obtained from XPS analysis. The XPS spectrum of cerium is very complex that contains multiple peaks from the spin orbit coupling of 3d orbitals [26]. Several peaks in the Ce3d region that have been ascribed to 3d_3/2_ (899.5, 900.9, 903.5, 906.4 and 916.6) and 3d_5/2_ (880.2, 882.1, 8885, 888.1 and 898) arising from multiple valence states of cerium. The spectrum from NCe shows a predominance of cerium in +3 oxidation state as depicted by the characteristic peaks at 880.2, 885.0, 899.5 and 903.5 eV. Taken together the data from characterization of NCe is consistent with previous reports wherein cerium can be retained in trivalent oxidation by decreasing the size of the nanoparticles [15], [16], [17], [26].

### NCe Treatment Inhibits Production of ROS Levels in A2780 Cell Line

Cerium oxide nanoparticles have been shown to act as free radical scavengers by inhibiting the production of reactive oxygen species (ROS) [3], [4], [5]. Since, it is well established that ROS accumulation plays an important role in the initiation and progression of tumorigenesis in human ovarian cancer [27], [28], [29], we examined the effect of NCe on ROS generation in ovarian cancer cell line A2780. A2780 cell line was treated with NCe (50–100 µM) and post 48 h of treatment, ROS generation was measured using DCFH2-DA dye followed by fluorescence reading. As shown in figure 1E–F, NCe treatment significantly inhibited ROS levels in A2780 cell line, suggesting that NCe treatment inhibits basal levels of oxidative stress in A2780 OvCa cell line.

### NCe did not Effect Cell Proliferation in Ovarian Cancer Cell Lines

**Figure 2:**
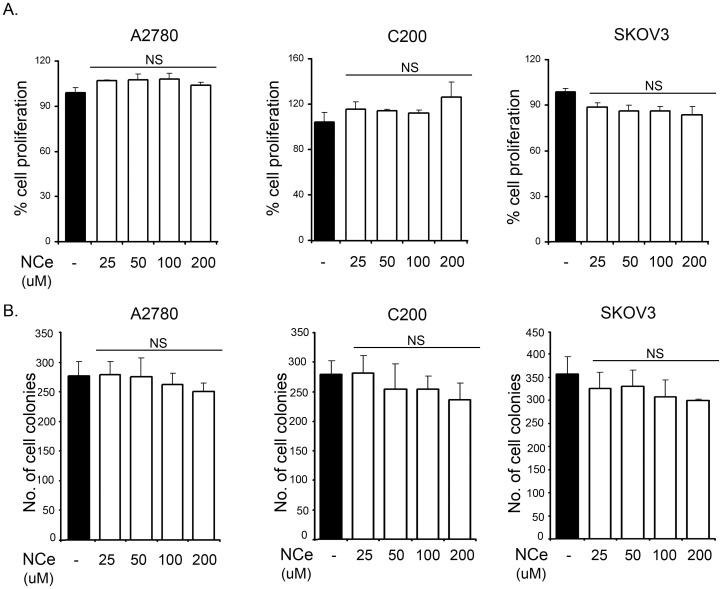
NCe treatment did not affect proliferation and colony formation in ovarian cancer cell lines. **A.** Percent viability of A2780, C200 and SKOV3 cells treated with indicated doses of NCe (25–200 µM) as determined by MTT assay. The data is represents three individual experiments done in triplicates. NS: not significant, compared to untreated cells at respective time point using two-tailed Student’s t-test (Prism). **B.** For colony formation, 2000 cells/well (A2780, C200 and SKOV3) were plated in 6-well plates and treated with indicated concentrations of NCe, every third day for 2 weeks until colonies were formed. The colonies were stained with MTT and counted. The data represents three separate experiments done in triplicates. NS: not significant compared to untreated cells using two-tailed Student’s t-test (Prism).

To determine if NCe treatment inhibited cell proliferation, A2780, C200 and SKOV3 were plated in 96 well plates at 4×10^3^ cells/well and treated with various concentrations of NCe (25–200 µM). Cell viability was determined at 72 hrs by MTT assay. As shown in figure 2A, NCe treatment had no significant effect on the proliferation or survival of ovarian cancer cell lines. Consistent with this observation, our clonogenic assays also showed no change in proliferation rate with increasing NCe concentration (Fig. 2B). [3H] thymidine uptake in A2780, C200 and SKOV3 cells (Fig. S1), also confirmed that NCe treatment did not alter the rate of proliferation. Similar results were obtained in additional ovarian cancer cell lines including CaOV3 and TOV21G (data not shown). Collectively, these results indicate that NCe treatment did not significantly inhibit the growth of ovarian cancer cells lines *in vitro*.

### NCe Inhibited Growth Factor-mediated Cell Migration and Invasion *in vitro*

**Figure 3:**
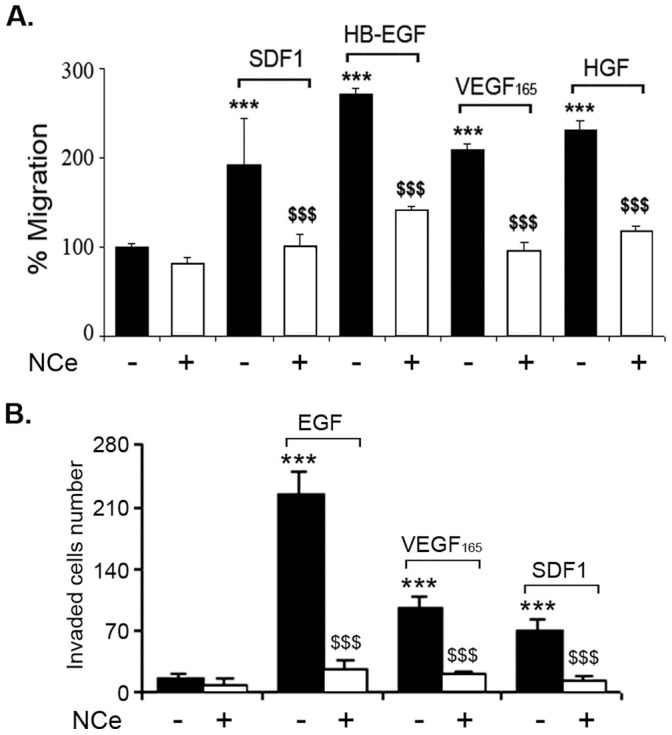
NCe treatment attenuated cell migration and invasion in ovarian cancer cell. **A.** Effect of NCe on various growth factors mediated cell migration was examined using wound closure assay as described in Material and Methods. Results are shown as mean ± S.D. of n = 7. **B.** To examine effect of NCe on invasion, 1×10^5^ SKOV3 cells were seeded into the upper wells with 100 µM NCe in the FIA chamber in 500 µl culture medium. Various growth factors (EGF, VEGF_165_ and SDF1) (25 ng/ml) was added to the underlying media. 24 hrs later, invasion was determined following manufacturer’s instructions. Results are shown as mean ± S.D. of triplicates. ***p<0.001 growth factor treated compared to control. $$$p<0.001 NCe treated compared to growth factor using two-tailed Student’s t-test (Prism).

Since, NCe treatment did not affect cell proliferation, we evaluated the effect of NCe on growth factor mediated cell migration and invasion. SKOV3 cells grown overnight in low serum (0.2%) containing medium were scratched using a sterile 200 µl pipette tip, once they reached 90% confluence. Various growth factors including SDF1, HB-EGF, VEGF_165_ and HGF (25 ng/ml) were added individually to the medium in the presence or absence of NCe (100 µM). 24 hrs later, the rate of wound closure was calculated. As shown in figure 3A, NCe inhibited all growth factor mediated cell migration in SKOV3 cell line. Similar results were obtained for A2780 and C200 cell lines (data not shown). Next we assessed the effect of NCe in inhibiting GF-mediated invasion of SKOV3 cells using Boyden chamber migration assay (BD Bioscience) as previously described [18]. Figure 3B clearly demonstrates that 100 µM NCe treatment significantly inhibited all growth factor induced invasion of SKOV3 cells compared to untreated cells. These results indicate that, NCe has the ability to inhibit migration and invasion of ovarian cancer cells without affecting cell proliferation.

### NCe Treatment Attenuated Tumor Growth in Human A2780 Ovarian Carcinoma Cell Line Bearing Nude Mouse Model

**Figure 4:**
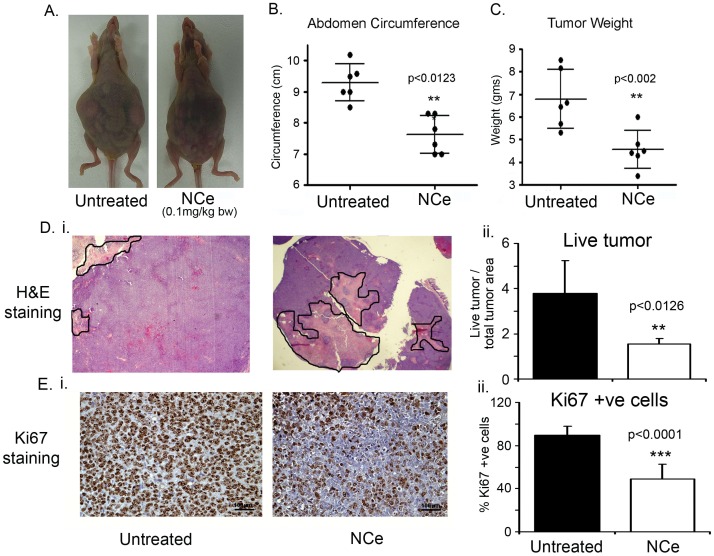
NCe treatment inhibited ovarian tumor growth *in vivo*. **A.** Gross morphology of representative mouse with tumors at day 30 (n = 6). **B.** Cumulative abdominal circumference at the end of the study. **C.** Excised tumor weight from vehicle (PBS) treated and NCe (0.1 mg/kg bd wt; every third day). Results are shown as mean ± S.D. of six individual animals. **p<0.01 NCe treated compared to untreated group using two-tailed Student’s t-test (Prism). **D.** **(i)** Representative H&E (x20) photomicrographs exhibiting live (purple) and necrotic (pink, encircled) areas in untreated and treated xenografts. **(ii)** Graphical representation of viable tumor size measured as described in Material and Methods. **E. (i)** Representative Ki-67 staining (x200) of excised A2780 xenografts at day 30. **(ii)** Count of positive Ki-67 cells from 5 high powered fields (x400) in 3 different xenografts from each group. Counts are expressed as percentage of control. ***p<0.001 and **p<0.01 NCe treated compared to untreated using two-tailed Student’s t-test (Prism).

To determine the effect of NCe *in vivo*, nude mice were intra-peritoneally injected with A2780 cells. Mice were treated with NCe (0.1 mg/kg body weight) given intra-peritoneally, every third day till end of the study (day 30) as described in the material and methods. Figure 4A (top panel) shows a representative photograph of a NCe treated and untreated mouse bearing A2780 xenograft at the end of study (day 30). The abdominal circumference, indicative of the tumor burden in the peritoneum (Fig. 4B) and the tumor weight (Fig. 4C) in the NCe treated mice were significantly (p<0.002) reduced compared to untreated mice. The mean weight of the excised tumors was approximately 33% less in NCe (4.56+0.345 gm) treated mice compared to vehicle (PBS) mice (6.79+0.53 gm) (Fig. 4C). This data clearly indicates that NCe has the ability to restrict ovarian tumor growth *in vivo* when administered even at a very low dose (0.1 mg/kg).

Representative H&E staining of A2780 xenografts (Fig. S2 -20×) showed more live cells in untreated xenografts. However, NCe treated xenografts had significantly more necrotic areas (dead cells) (Fig. 4Di; 20×; non-viable area encircled). To quantitate the live and dead areas in the xenograft sections, uni-dimensional measurements of the viable tumor and total tumor size were taken as described in the methods section. NCe treated tumors had less viable tumor areas than untreated mice (Fig. 4Dii). To determine whether the decrease in tumor weight and size following NCe treatment was due to decreased cell proliferation, immuno-histochemical analysis of Ki67 was performed. Significant difference was observed in the number of cells staining positive for Ki-67 (Fig. 4Ei). Enumeration of Ki-67 positive cells counted over 5 high power fields of five sections from each group also showed significant less Ki-67 positive cells in NCe treated xenografts compared to untreated group (expressed in %age; Fig. 4Eii), indicating that less number of cells were proliferating under NCe treatment. Together, these data indicate that NCe has the ability to restrict ovarian tumor growth *in vivo* due to decreased proliferation of OvCa cells.

**Figure 5:**
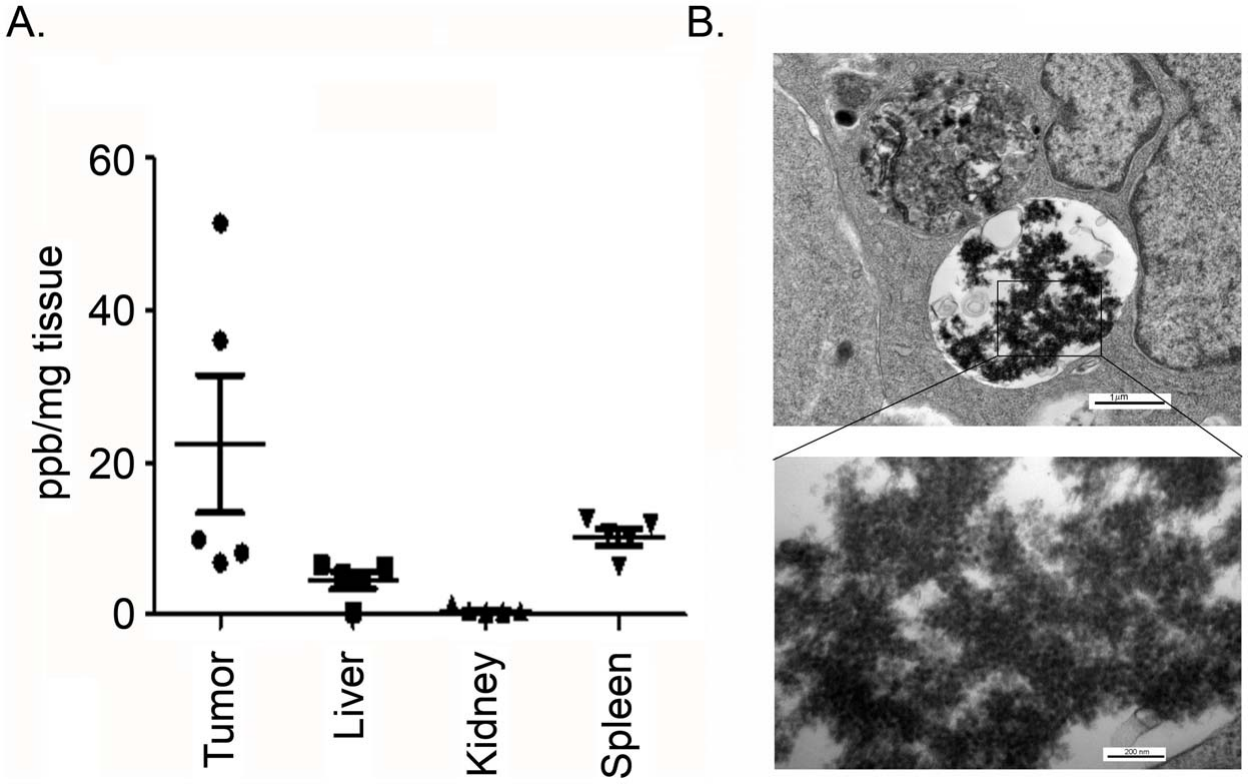
Bio-distribution of NCe in ovarian tumor bearing nude mice. **A.** At the end of the study, various organs including tumor, liver, kidney and spleen were isolated from five individual mice and tissues were digested using microwave as described in method. Level of NCe was measured using ICP-MS. Data represents mean+SEM of 5 tissues of individual mouse. **B**. Agglomeration of NCe localized in vacuole in ovarian cancer tumor examined by TEM as described in Method in detail.

To examine if NCe is accumulated in tumors, at the end of the study, tumors were isolated along with major organs including liver, kidney and spleen from NCe treated groups. NCe was isolated and detected using ICP-MS as described in the methods. As shown in figure 5A, high levels of NCe could be detected in tumors followed by spleen and liver levels. Least amount of NCe was detected in kidney. To further strengthen this observation, we performed electron microscopy in tumors isolated from NCe treated mice and found the agglomeration of NCe in tumor tissue, further suggesting that NCe is accumulated in tumor and may be one of the reasons for decreased tumor burden in treated mice compared to vehicle treated group.

**Figure 6:**
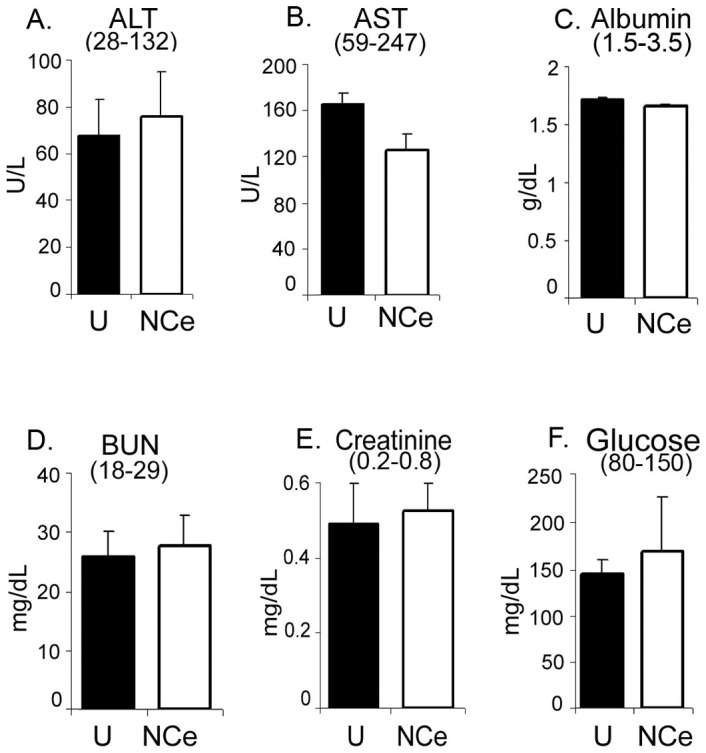
NCe treatment is non-toxic in nude mice. After sacrificing animal groups (n = 6) at the end of the study (day 30), blood was collected in heparin coated tubes and plasma was prepared. A panel of liver and kidney cytotoxic tests were performed in plasma prepared from control and NCe treated mice as per the manufacturer’s instructions, **A.** ALT (Alanine Transaminase), **B.** AST (Aspartate Transaminase), **C.** Albumin, **D.** BUN (Blood Urea Nitrogen), **E.** Creatinine and **F.** Glucose.

To determine the cellular toxicity of NCe *in vivo*, the vital organs (liver heart, spleen, kidney and lungs) were excised from untreated and NCe treated mice at the end of the study and formalin fixed. H&E staining of the various sections revealed that the morphology of all organs from treated and untreated mice appeared to be normal and no necrosis was observed (Fig. S3A-E; 100×). Analysis of liver function (aspartate aminotransferase, AST; alanine aminotransferase, ALT; albumin) and kidney function (creatinine; blood urea nitrogen, BUN; albumin) in plasma collected, showed no significant difference in the untreated and NCe treated mice. All values were found within the normal limits in both groups (Fig. 6A–E). No fluctuation in the glucose levels was observed either (Fig. 6F). These data show that NCe treatment on every third day for 4 weeks at the dose of 0.1 mg/kg is safe and does not result in tissue cytotoxicity or any abnormal physiological vital functions.

### NCe Inhibited Metastasis in Human A2780 Ovarian Carcinoma Cell Line Bearing Nude Mouse Model

**Figure 7:**
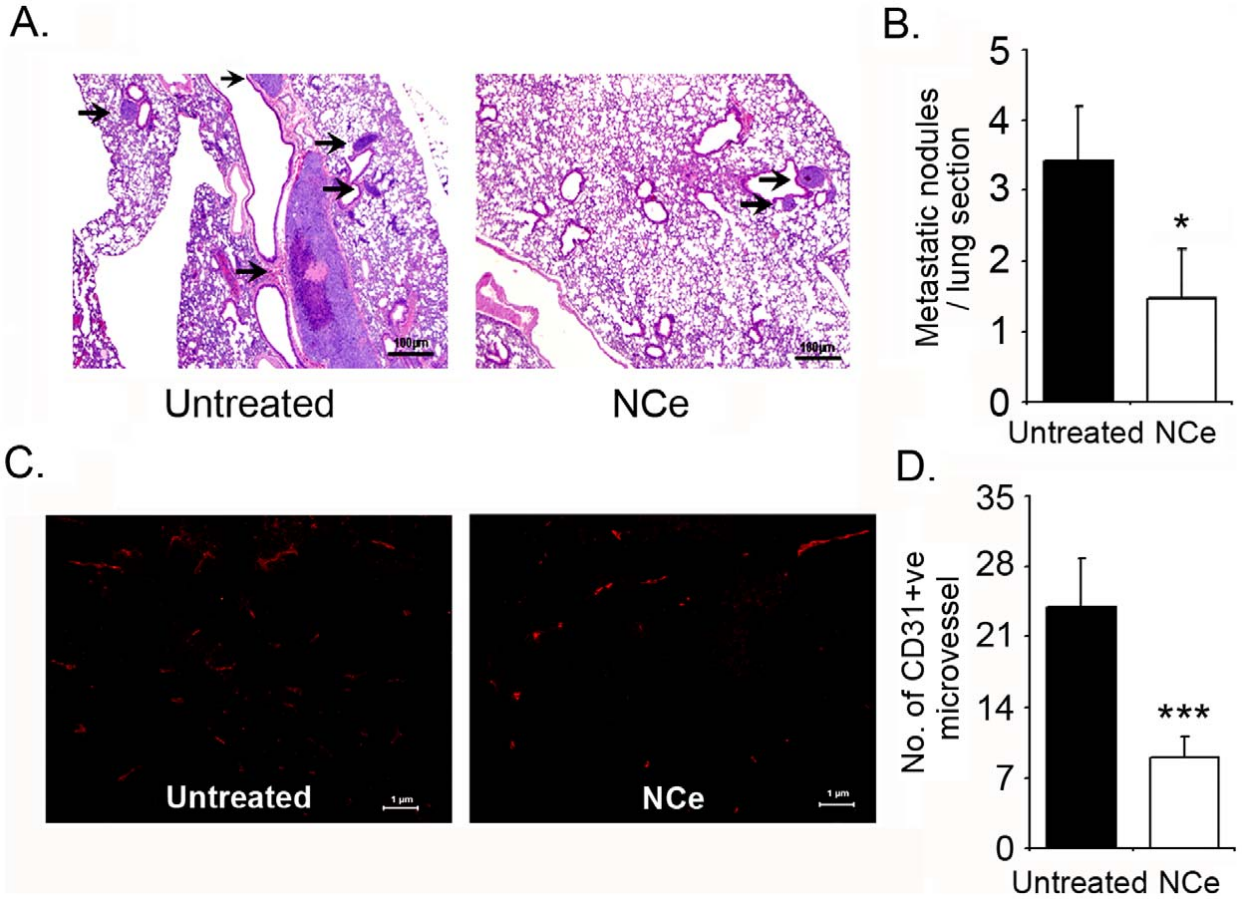
NCe inhibits metastasis and vessel formation in A2780 xenografts. **A.** Representative H&E (x100) photomicrograph depicting lung metastatic nodules from Untreated and NCe treated mice (arrows point to metastatic nodules). **B.** Enumeration of metastatic nodules found per lung from Untreated and NCe treated mice. Total of 5 lung sections were observed to get the average count. *p<0.005 NCe compared to untreated using two-tailed Student’s t-test (Prism). **C.** Representative photomicrograph of CD31 staining of blood microvessels (x200) in A2780 xenografts at day 30. **D.** Count of average microvessels per high power field (x400) from five fields of three xenograft section from untreated and NCe treated mice. ***p<0.001, NCe treated to untreated using two-tailed Student’s t-test (Prism).

Since our *in vitro* data showed that NCe treatment inhibited growth factor induced migration and invasion, we further examined for the presence of metastasis in various organs. H&E staining of sections from various organs revealed no invasion of tumor cells inside liver, spleen and kidney (data not shown), although tumor nodules were visible on their surface in untreated mice. However, large metastatic nodules were observed in lungs of untreated mice, which were significantly reduced in lungs of NCe treated mice. Figure 7A (400×). More importantly, the number of nodules were also significantly decreased upon NCe treatment (Fig. 7B). These results are consistent with our *in vitro* data where, NCe treatment inhibited migration and invasion of tumors cells *in vitro*.

### NCe Inhibited Angiogenesis in A2780 Ovarian Carcinoma Mouse Model

As we did not observe any significant effect on cell proliferation or cell death in ovarian cancer cell lines *in vitro* but found significant reduction in tumor growth *in vivo*, we hypothesized that this could be a result of inhibition of angiogenesis as it is one of the important factors implicated in progression of OvCa [30], [31]. Also for a tumor to sustain, grow, proliferate and invade, formation of new vasculature is a prerequisite. Micro-vessel density of tumors increases in response to various angiogenic factors and low oxygen availability in the growing tumors, which enables them to avoid death and proceed towards invasion and metastasis. We evaluated the micro-vessel density by staining for CD31. CD31 staining of xenograft sections from NCe treated A2780 tumors showed significantly less number of CD31 positive micro-vessels (Fig. 7C, 200×) compared to untreated mice. Quantification of positively stained vessels at high power from 5 different fields of five sections from each group is shown in figure 7D, 400×. These indicate that NCe maybe targeting angiogenesis and as a consequence limiting tumor growth.

### NCe Treatment Targets Angiogenesis by Inhibiting VEGF Signaling in Endothelial Cells

VEGF_165_, one of the most potent angiogenic factor is overexpressed in ovarian tumors [32], [33], [34]. To determine if NCe treatment could be attenuating angiogenesis by inhibiting VEGF production/levels, we initially examined the effect of NCe on VEGF_165_ production in SKOV3 cells *in vitro*. SKOV3 cells were grown, kept in serum free conditions overnight and treated with EGF to stimulate VEGF production. Post 24 h, conditioned media was used to estimate the levels of VEGF by ELISA. As shown in Fig. S4A, treatment with EGF induced the production of VEGF in SKOV3 cells, while no changes were found in VEGF levels when cells were treated with NCe. Under similar experimental conditions we also estimated the levels of IL-8, which is involved in OvCa pathogenesis and VEGF production [35]. Similar to VEGF, NCe did not alter the levels of IL-8 production (Fig. S4B). Consistent with this observation, immunostaining for VEGF_165_ levels in the A2780 xenografts from NCe treated and untreated mice also showed no change in the VEGF expression (Fig. S4C). Collectively, these findings clearly suggest that NCe does not modulate VEGF and IL-8 levels in ovarian cancer cells but restricts angiogenesis by some other mechanism.

**Figure 8:**
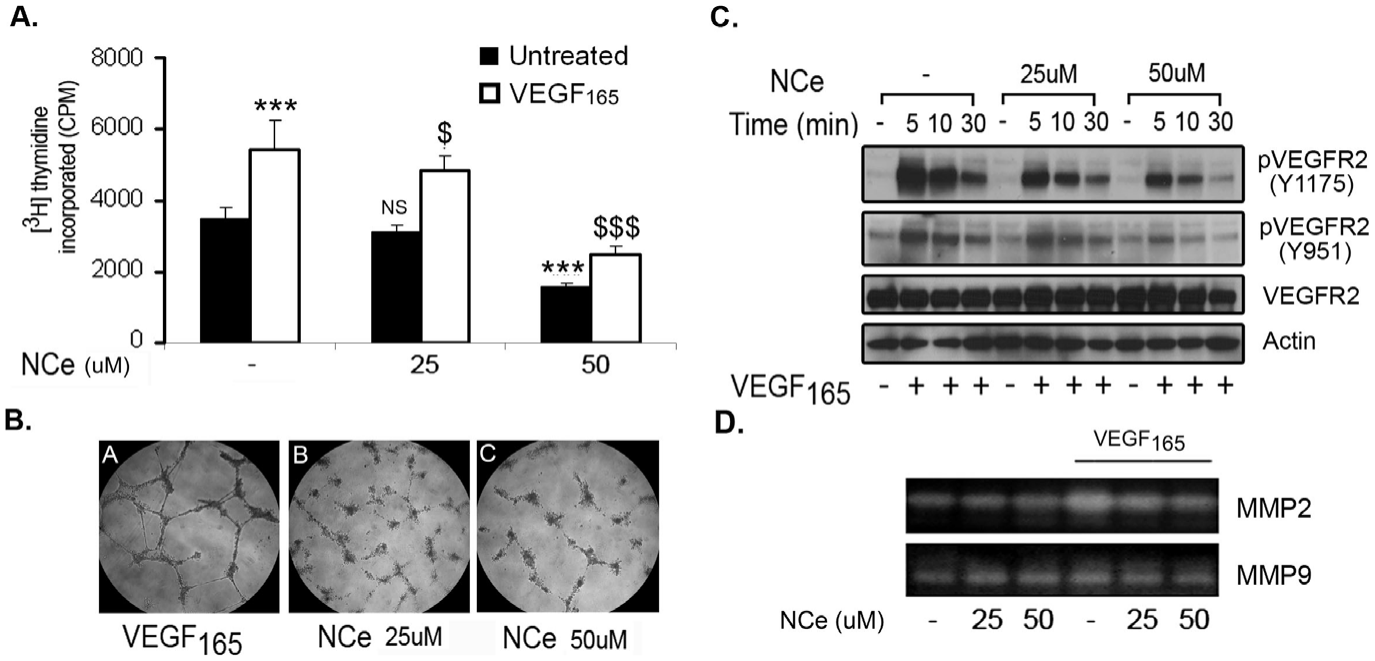
NCe treatment inhibits VEGF _165_ induced downstream signaling in endothelial cells. **A.** NCe inhibited VEGF_165_ mediated proliferation of HUVEC cells as assessed by measurement of DNA synthesis by [3H]-thymidine incorporation (n = 4). ***, *p*<0,001; NS not significant compare with untreated cells. $, *p*<0.05; $$$, *p*<0,001 compared with VEGF_165_ treated cells using two-tailed Student’s t-test (Prism). **B.** The effect of NCe on tube formation in HUVEC cells was examined as described in Method and Materials. **C** NCe treatment inhibited VEGF_165_ induced phosphorylation of VEGFR2 (Y1175 and Y951), as seen by immunoblot. **D.** NCe inhibited VEGF_165_ induced MMP2 activity in HUVEC without affecting MMP9 activity examined by zymography.

Since NCe treatment significantly reduced the number of microvessels in treated xenografts without affecting the VEGF expression, we surmised that NCe may modulate angiogenic signaling downstream of VEGF_165_ in endothelial cells. NC e treatment attenuated VEGF_165_ mediated proliferation of HUVEC cells as determined by thymidine incorporation assay (Fig. 8A). The next step for proliferating endothelial cells is to form new vessels. HUVEC cells (2×10^4^) were plated on matrigel coated 96 well plate in the presence of NCe (25–50 µM) with or without VEGF_165_ (25 ng/ml) as an angiogenic stimuli. VEGF_165_ induced endothelial tube formation was significantly attenuated by NCe treatment (Fig. 8B). NCe treatment also reduced the phosphorylation of VEGFR2 at Tyr1175 and Y951in HUVEC cells in response to VEGF_165_ treatment (Fig. 8C). Additionally**,** NCe treatment also inhibited VEGF induced MMP2 activity in endothelial cells as determined by zymography assay (Fig. 8D). These data clearly suggest that NCe treatment inhibits VEGF mediated downstream signaling in endothelial cells and as a result may interfere with proliferation and survival of endothelial cells.

### NCe Treatment Specifically Targets Endothelial Cells *in vivo*

**Figure 9:**
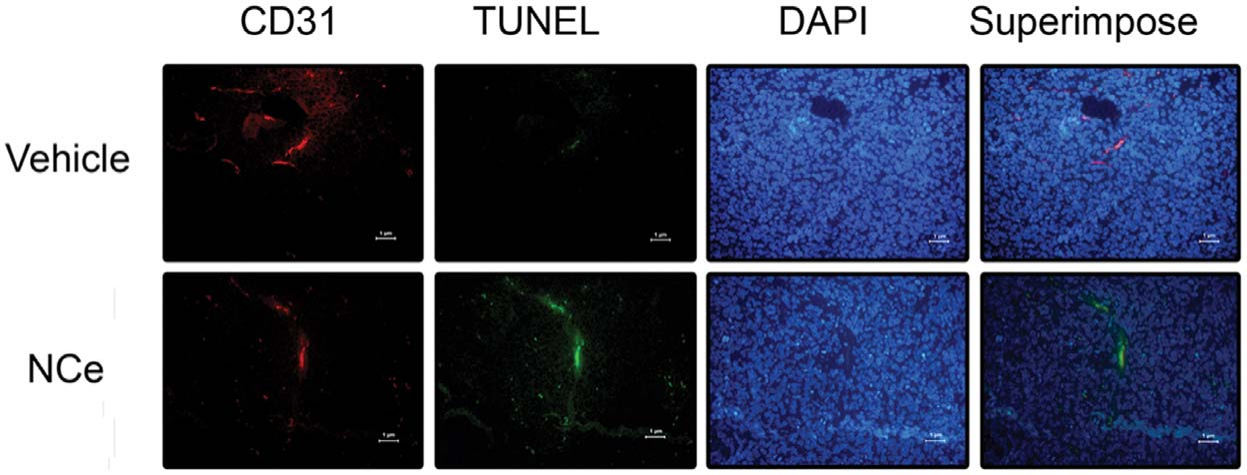
NCe treatment potentiates the endothelial apoptosis of ovarian tumor *in vivo*. Double of staining CD31 and TUNEL was performed as described in Methods. First panel depicts CD31-FITC labeled endothelial cells as part of a microvessel (red, x400). Second panel depicts the same microvessel stained positive for apoptotic marker TUNEL labeled with FITC (green, x400). Third panel shows blue nuclei stained with DAPI (x400), indicating presence of tumor cells. The last panel shows merged image of all first three panels. The microvessel appears yellowish due to co-localization of CD31 and TUNEL in NCe treated tumor section.

To determine if NCe has any inhibitory effect on vessel formation *in vivo* as observed *in vitro*, we performed double staining with CD31 and TUNEL in the tumor xenograft slides from both treated and untreated mouse groups. As shown in Fig. 9, CD31 antibody recognized endothelial cells in the microvessels (stained red, first panel). Cells undergoing apoptosis were detected by performing TUNEL staining (stained green, second panel). Upon superimposing, it was observed that TUNEL and CD31 stains were co-localized (yellow, last panel), indicating that it was the endothelial cells specifically in the microvessels that were undergoing apoptosis under NCe treatment. These data suggest a potential role for NCe as an anti-angiogenic molecule by targeting endothelial cells and VEGF signaling.

## Discussion

The science of developing nanoparticles into nano-medicine to encounter human diseases for better health outcomes is a rapidly progressing field. A number of metal nanoparticles have been designed and shown to be of therapeutic interest in various animal models, especially in the field of cancer [36], [37]. Successful incorporation of nanoparticles as anti-cancer therapeutics can open an entirely new avenue for cancers like ovarian, where chemotherapeutic options are limited and high mortality is a serious concern. In this regard, we investigated the potential of a specially designed cerium oxide nanoparticles, (nanoceria; NCe) as a therapeutic agent in ovarian cancer.

In the present study, we show for the first time that NCe has the potential to inhibit ovarian tumor growth and metastasis. We show that NCe attenuated basal levels of oxidative stress, invasion and migration of ovarian cancer cells without modulating their cell growth. It also significantly attenuated tumor growth in A2780 bearing nude mice when given intra-peritoneally. Our study found a novel property of NCe as an anti-angiogenic as its treatment reduced the microvessel density in ovarian xenografts, inhibited proliferation and induced apoptosis in endothelial cells *in vitro* and *in vivo* respectively. Additionally, it also attenuated VEGF mediated downstream signaling in HUVEC. *In vivo* treatment of NCe resulted in specific apoptosis of endothelial cells in the microvessels being formed in the tumor tissue. Overall, our study presents a novel attribute of NCe as an anti-angiogenic agent, which can be used as a therapeutic in OvCa and other cancers.

The most attractive property of cerium oxide nanoparticles is their capacity to serve as free radical scavengers to provide protection against chemical, biological, and radiological insults that promote the production of free radicals [3], [4], [5]. NCe offers many active sites for free radical scavenging due to its large surface/volume ratio and mixed valence states (+4 and +3) for unique redox chemistry. Moreover, its unique regenerative property (Ce3+-Ce4+-Ce3+) [4] makes NCe long-lived and can thus confer its beneficial effect for extended periods of time without limiting the number of frequent dosage. Recently, it has been reported that NCe selectively conferred radioprotection to the normal breast cells (CRL 8798) against ROS compared to the breast cancer cells [5]. It also provided radioprotection against pneumonitis and gastrointestinal epithelium by reducing ROS [38], [39]. Another recent study showed NCe to bestow protection from monocrotaline-induced hepatoxicity due to oxidative stress [40]. NCe has been shown to induce oxidative stress in other cancer cell lines including human bronchoalveolar carcinoma derived cell line (A549) and squamous SCL-1 tumor cell line [41]. Our finding that NCe acts as an anti-oxidant in ovarian cancer, is in agreement with these previous reports. These data suggest that cerium oxide particles may have differential outcomes of its anti-oxidant properties dependent on the nature of cell type.

In our study, although the anti-oxidant property of NCe was able to reduce basal levels of oxidative stress but had no effect on proliferation of OvCa cell lines *in vitro.* Interestingly, NCe inhibited growth factor induced migration and invasion of ovarian cancer cell lines. Similar to our observation, Tarnuzzer et al [5] also found non-cytotoxic effect of NCe on MCF7 breast cancer cell line. However, polymer-coated NCe showed cytotoxic effect on squamous tumor SCL-1 cells [41]. The discrepancy between ours and other [41] reports may be due to subtle change in the surface properties of nanoparticles in various cell types.

On observing that NCe treatment inhibited GF induced migration/invasion *in vitro*, we examined its ability to modulate tumor growth *in vivo*. We found that administration of NCe (0.1 mg/kgbdwt) every third day significantly retarded A2780 xenograft growth *in vivo* which was also accompanied by attenuation of metastatic nodule size and number in lung. This is the first report that demonstrates the *in vivo* ability of NCe to inhibit ovarian tumor growth and metastasis. NCe accumulated mainly in tumor followed by spleen, liver and then kidney when given to tumor bearing mice. Uptake of NCe in tumor was further confirmed by TEM where we were able to detect agglomeration of NCe. Also, used dose of NCe (0.1 mg/kgbdwt) is well tolerated by mice without any cytotoxic or adverse physiological effects observed in the vital organs. These findings are in agreement of previous report where NCe at a high concentration of 135 mg/kg body weight did not cause any death or notable side effect in nude mice with normal pathology of vital organs [38]. However, a recent report indicates the toxic effect of NCe in rodents [42]. This can be accounted for as the dose of NCe used in the previous study is 1000–2500 times higher (100–250 mg/kg body weight) then what we have used in our study (0.1 mg/kg body weight) [42].

Although *in vitro*, NCe treatment did not affect ovarian cancer cell growth *in vitro*, we observed decreased staining of Ki-67, a maker of cell proliferation *in vivo* in the xenografts, suggesting NCe treatment might have an effect on the tumor micro-environment rather than having a direct effect on the tumor cells. To determine the mechanism by which NCe maybe restricting tumor growth, we examined the microvessel density in treated and untreated tumor tissue and found a significant reduction in NCe treated tumors as evident from CD31 staining, a marker for endothelial cells. Interestingly, NCe did not modulate VEGF production *in vitro* and *in vivo*. These observations led us to hypothesize that NCe maybe specifically targeting the endothelial cells responsible for formation of new vessels.

Angiogenesis is important for tumor development and growth and VEGF has been shown to be a major angiogenesis inducer primarily through the VEGF type 2 receptor (VEGFR2) [43], [44]. The downstream effects of VEGF mediated signaling include increase in cell proliferation, tube formation and activation of matrix metalloproteases to promote degradation of extracellular matrix. We observed that NCe treatment inhibited VEGF induced proliferation, capillary tube formation and MMP2 activation in endothelial cells. NCe treatment attenuated VEGF mediated phosphorylation of VEGFR2 (Y1175 and Y951), a prerequisite for VEGF to signal to endothelial cells. Phosphorylation of VEGFR2 (Y1175) is critical for endothelial proliferation and the recruitment of adaptor proteins including p85 of PI3Kinase and PLCγ [45]. Phosphorylation of VEGFR2 at Y951 is important for recruitment of adaptor proteins [46]. Inhibition of VEGF induced downstream signaling including proliferation, tube formation and MMP2 activation by NCe treatment indicating a novel anti-angiogenic property of NCe. Targeting of endothelial cells by NCe *in vivo* was also supported by the TUNEL assay in NCe treated xenografts specific to CD31+ve endothelial cells. The role of oxidative stress in angiogenesis is an emerging area of investigation [47]. The anti-angiogenic property of NCe might be modulating VEGF mediated signaling events which are redox sensitive [44]. This is the first report demonstrating any cerium oxide nanoparticles to exhibit an anti-angiogenic property with specificity for endothelial cells.

Anti-angiogenic agents have demonstrated activity in terms of both response and progression free survival (PFS) in phase II and phase III clinical trials for women with epithelial ovarian cancer, both in the front-line and relapsed setting [48], [49], [50], [51]. But recent emerging reports indicate the therapy to be associated with increased fatal reactions [52], [53], [54], [55]. Therefore, novel strategies and agents that can target angiogenesis without many side effects are still required.

Taken together, our results reveal a new role of cerium oxide nanoparticles as a novel, non-toxic anti-angiogenic agent that restricted ovarian tumor growth in preclinical mouse model of ovarian cancer. Our study opens up a new avenue of using nanoparticles as feasible therapeutics in ovarian cancer. A future option to increase the efficacy and specificity of NCe, can be conjugating it with folic acid, a ligand for folate receptor over expressed in ovarian cancer, as a tool to deliver NCe specifically to tumor as a novel targeted therapy. Moreover, NCe could be conjugated with other chemotherapeutic drugs for their specific delivery as well as enhancement of their cytotoxicity, while reducing the side effects. The outcome of these studies could open a new and novel avenue of therapy for ovarian cancer.

## Supporting Information

Figure S1 **NCe has no effect on cell proliferation of ovarian cancer cell lines.** [^3^H]Thymidine incorporation following NCe treatment in **A.** A2780, **B.** C200 and **C.** SKOV3 shows that NCe treatment has no effect on proliferation of ovarian cancer cells. The data is representation of three separate experiments done in triplicates. NS, non-significant compared with control using two-tailed Student’s t-test (Prism).
(TIF)

Figure S2 **Representative photomicrograph of H&E staining (200×) of A2780 xenografts at day 30.**
(TIF)

Figure S3 **NCe treatment is non-toxic in nude mice bearing human A2780 carcinoma.** After sacrificing animal groups, different organs of five mice from each group were formalin fixed, processed for histological sectioning and stained with H&E to observe morphology of the tissue. Representative photomicrographs (100×) of **A.** Liver; **B.** Heart; **C.** Spleen; **D.** Kidney and **E.** lungs, show normal morphological architecture in tissues of both untreated and NCe treated mice.
(TIF)

Figure S4 **NCe does not affect the production of VEGF and IL8 in SKOV3 cells.** SKOV3 cells were plated and kept under serum free conditions overnight before being stimulated by EGF (10 ng). Post 24 h supernatant was collected to perform ELISA. **A.** VEGF levels. **B.** IL-8 levels. *p<0.01, **p<0.001 of EGF treated to no treatment. NS = non-significant NCe treated to untreated; ns = non-significant NCe/EGF treated to EGF treated using two-tailed Student’s t-test (Prism). **C.** Representative photomicrograph of VEGF staining (400×) in A2780 xenografts at day 30.
(TIF)
