# Supplementary figures and images for: Nanoceria: A Rare-Earth Nanoparticle as a Novel Anti-Angiogenic Therapeutic Agent in Ovarian Cancer
Source: PLoS One. 2013 Jan 31;8(1):e54578. doi: 10.1371/journal.pone.0054578 (PMC3561344; doi:10.1371/journal.pone.0054578)

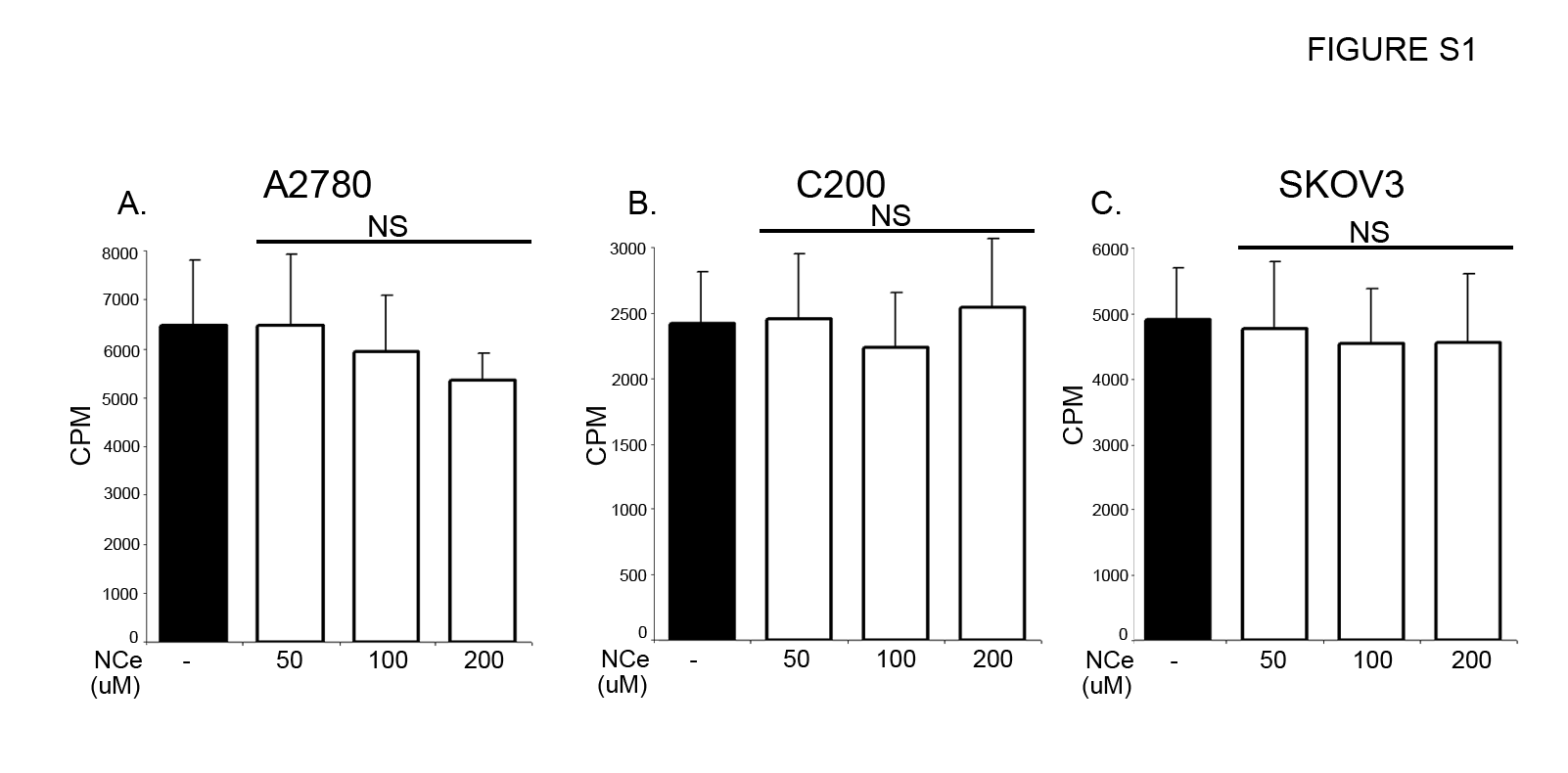

Supplement: Figure S1 — NCe has no effect on cell proliferation of ovarian cancer cell lines. [3H]Thymidine incorporation following NCe treatment in A. A2780, B. C200 and C. SKOV3 shows that NCe treatment has no effect on proliferation of ovarian cancer cells. The data is representation of three separate experiments done in triplicates. NS, non-significant compared with control using two-tailed Student’s t-test (Prism). (TIF) [file pone.0054578.s001.tif]

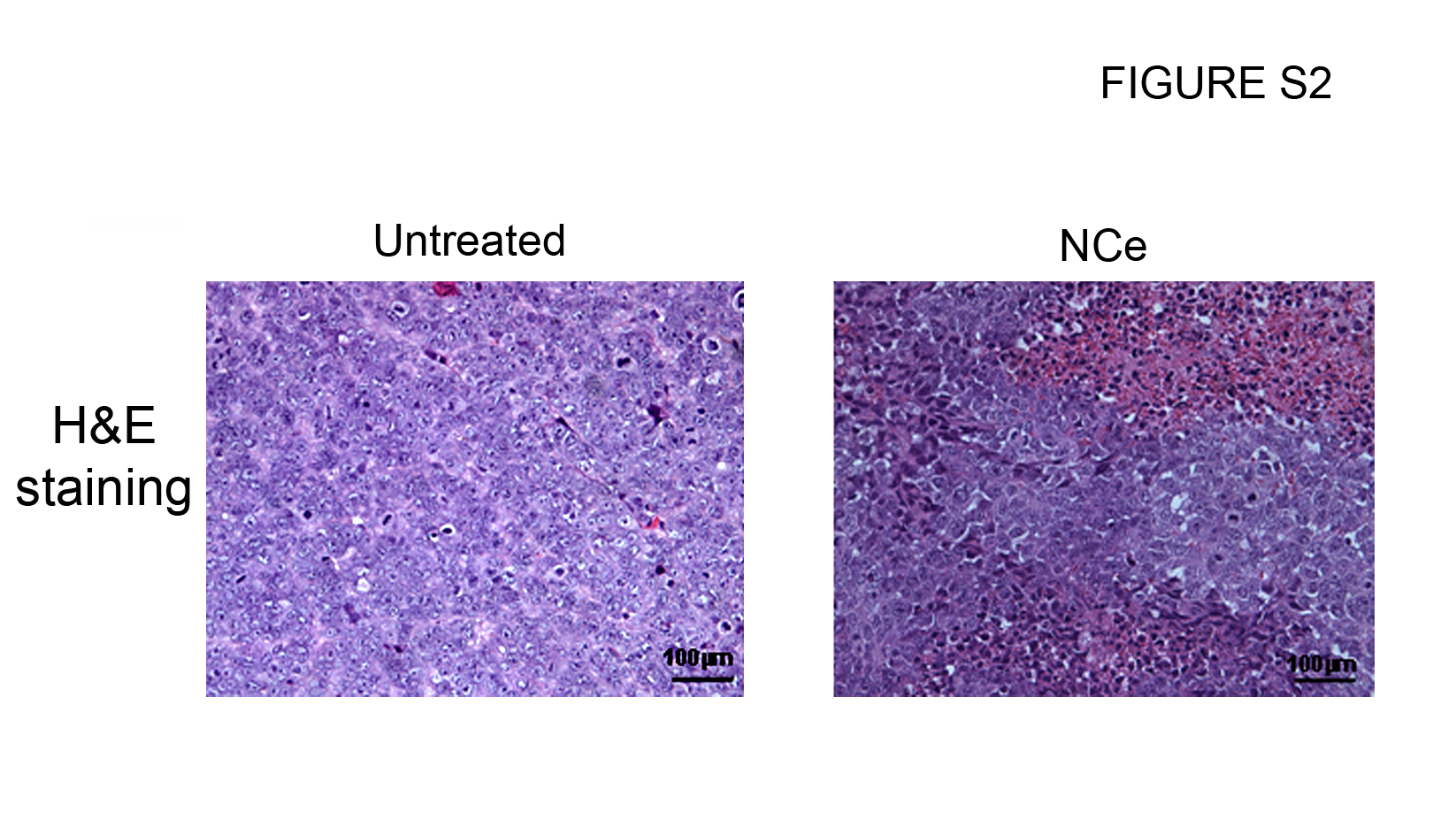

Supplement: Figure S2 — Representative photomicrograph of H&E staining (200×) of A2780 xenografts at day 30. (TIF) [file pone.0054578.s002.tif]

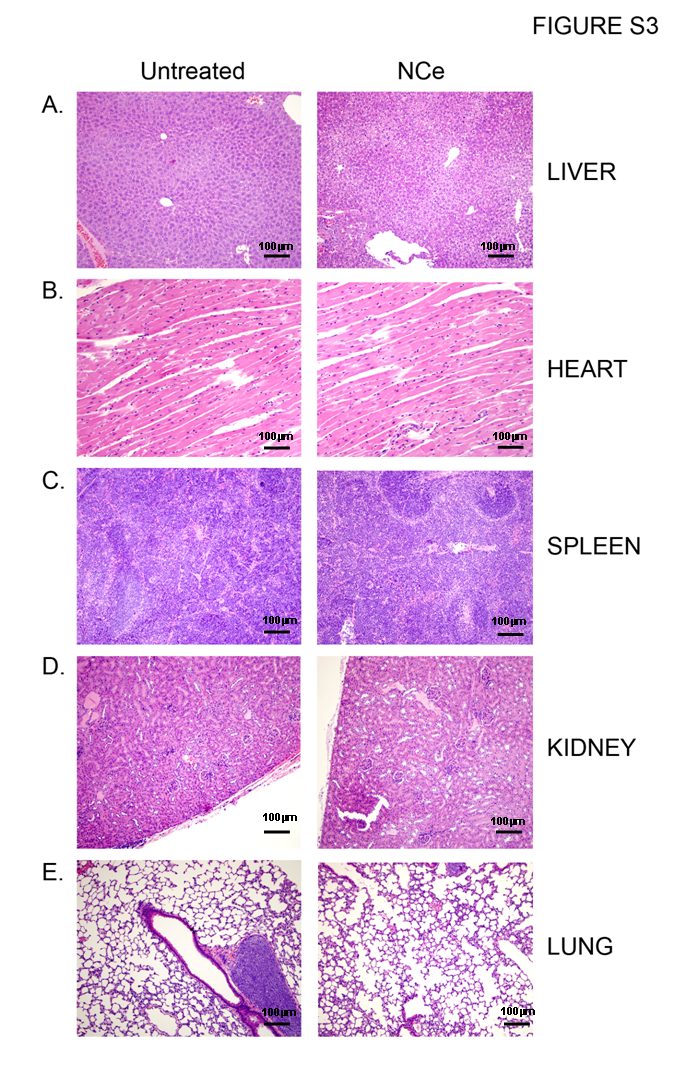

Supplement: Figure S3 — NCe treatment is non-toxic in nude mice bearing human A2780 carcinoma. After sacrificing animal groups, different organs of five mice from each group were formalin fixed, processed for histological sectioning and stained with H&E to observe morphology of the tissue. Representative photomicrographs (100×) of A. Liver; B. Heart; C. Spleen; D. Kidney and E. lungs, show normal morphological architecture in tissues of both untreated and NCe treated mice. (TIF) [file pone.0054578.s003.tif]

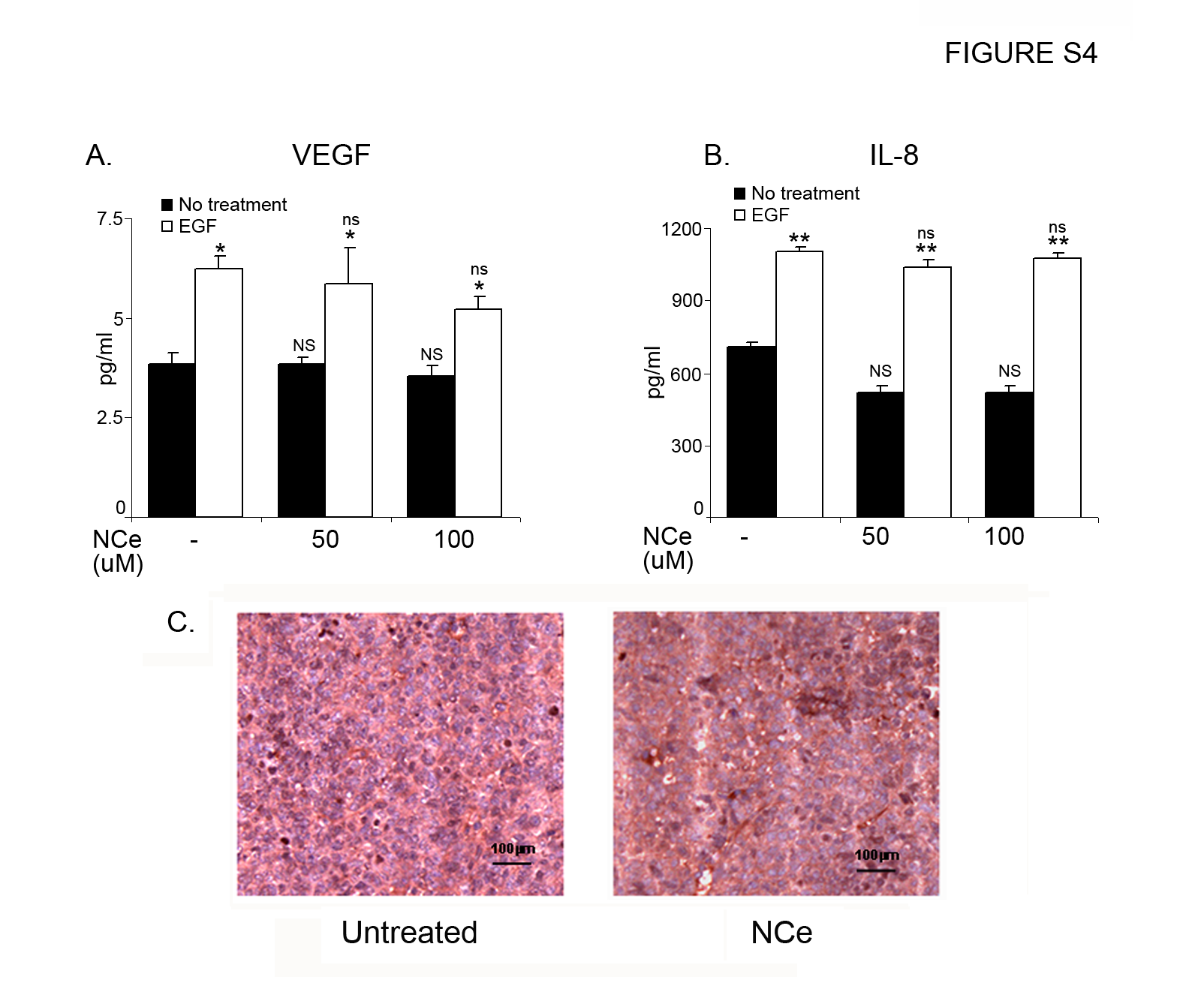

Supplement: Figure S4 — NCe does not affect the production of VEGF and IL8 in SKOV3 cells. SKOV3 cells were plated and kept under serum free conditions overnight before being stimulated by EGF (10 ng). Post 24 h supernatant was collected to perform ELISA. A. VEGF levels. B. IL-8 levels. *p<0.01, **p<0.001 of EGF treated to no treatment. NS = non-significant NCe treated to untreated; ns = non-significant NCe/EGF treated to EGF treated using two-tailed Student’s t-test (Prism). C. Representative photomicrograph of VEGF staining (400×) in A2780 xenografts at day 30. (TIF) [file pone.0054578.s004.tif]
